# Ultrasound vs. clinical diagnosis—which is better in diagnosing acute appendicitis?: a cohort study

**DOI:** 10.1097/MS9.0000000000000730

**Published:** 2023-05-04

**Authors:** Cheuk Tung Kam, Mahdi Alsahaf, Kul Chongbang, Mona Taki, Jamal Yusob, Santhosh Alexander, Jaideep Rait

**Affiliations:** a Department of General Surgery, Maidstone and Tunbridge Wells NHS Trust; b William Harvey Hospital, East Kent University Hospital NHS Foundation Trust; c Medway Maritime Hospital, Medway Foundation Trust; Departments of d Emergency Medicine; e Surgery, Maidstone and Tunbridge Wells NHS Trust, Kent, UK

**Keywords:** Acute appendicitis, clinical evaluation, negative appendicectomy, ultrasound

## Abstract

Acute appendicitis is one of the most common causes of right iliac fossa pain which often warrants surgical management. In many cases, abdominal ultrasonography (USS) is done to confirm the diagnosis however negative appendicectomy is common due to atypical presentations and different visualisation rate. The unnecessary operative intervention can result in complications. The aim of this study was to compare the efficacy between clinical diagnosis and USS in diagnosing acute appendicitis to avoid negative appendicectomy and prevent further complications.

**Method:**

A retrospective cohort study was conducted in a district general hospital which 1046 cases of laparoscopic appendicectomy were included and examined for the use of USS or clinical diagnosis. The sensitivity, specificity, negative and positive predictive value in USS and data in clinical evaluation were analysed for their accuracy in the diagnosis of acute appendicitis based on the histology results post appendicectomy.

**Results:**

Clinical diagnosis without preoperative imaging was found to have a significantly lower negative appendicectomy rate of 27.20% compared with 42.67% in those who underwent ultrasound. 44.64% of the patients were misdiagnosed with a normal USS result who had a subsequent positive histology of appendicitis.

**Conclusions:**

USS has been shown to be less sensitive for diagnosing acute appendicitis which results in high negative appendicectomy and misdiagnosis rate. With an increasing burden of health budget and resources, cautious and appropriate use of USS would avoid the misdiagnosis and prevent further complications. Thorough clinical evaluation remains an important first step and role in the diagnosis of acute appendicitis.

## Introduction

Highlights

- Clinical evaluation is an important first step in diagnosing acute appendicitis.
- Ultrasound should be utilised cautiously in diagnosing acute appendicitis.
- A combination of ultrasound and clinical review helps diagnosing acute appendicitis.

Acute appendicitis is one of the commonest referrals to the general surgical team^1^. Clinical diagnosis alone is sometimes challenging given to a range of presentations and potential pathologies, especially with other confounding factors such as ectopic pregnancy or other gynaecological pathology in female patients. As the outcome of a missed diagnosis can be disastrous, prompt diagnosis and management are essential for which appendicectomy can be justified by only clinical history, examination and basic laboratory investigations. However, unnecessary surgical intervention carries morbidity and complications^2^. Negative appendicectomy (NA) is common due to atypical presentations and different visualisation rate on ultrasonography (USS). According to an international and multicentre study, it has been proven that a fifth of the patients in the United Kingdom who underwent appendicectomy have a normal appendix on histology^3^.

USS is a noninvasive imaging modality and routinely utilised to aid the diagnosis of appendicitis^4^. Despite the advancement in modern radiological investigations, appendicitis can still be misdiagnosed by its high operator dependency. An ultrasonic diagnosis of appendicitis would be suggested by a blind ending, aperistaltic and incompressible tubular structure in the right iliac fossa with a thickened wall^5^.

The primary aim of this study was to compare the NA rate between clinical diagnosis and USS to avoid and prevent further complications. The secondary aim was to analyse the sensitivity, specificity, positive predictive value and negative predictive value of ultrasonography in diagnosing appendicitis and assess the role of this imaging study in its diagnosis.

## Methods

A retrospective single centre cohort study was conducted in a UK district general hospital over a 6-year period. Data from 1340 patients aged between 13 and 96 (median age 30) electronically coded as laparoscopic appendicectomy were extracted from an electronic theatre record. Two hundred ninety-four patients who had computerised tomography (CT) preoperatively were excluded. The data were then grouped into: appendicitis, normal or alternative pathology. USS were performed by registered sonographers and consultant radiologists all experienced in performing abdominal ultrasounds. Reporting system and examination technique were standardised for the patients who underwent ultrasound to reduce interoperator variability.Positive findings on USS as acute appendicitis include direct and indirect signs with graded compression technique. The results of preoperative USS (if performed) and the final histology for each case were analysed and compared. Ethical approval was not required as anonymised data were collected and the research was registered (UIN: researchregistry8805)^6^. This study followed the standards for STROCSS criteria^7^.

Data from a total number of 1046 patients was collected which satisfied the sample size of 248 patients per group (effect size 0.8, 95% confidence level and power of 80%)and examined using Microsoft Excel and statistical tests were run through the use of MedCalc softwareEvaluation of USS data was then performed through the use of the medical statistic software to obtain the sensitivity, specificity, negative predictive value and positive predictive value with 95% confidence level (Table 4). Data in clinical evaluation alone were also analysed (Table 2). Null hypothesis was established as there is no difference in the negative appendicectomy rate between the two patient groups and statistical tests were run with an unpaired *t*-test to determine the significance of preoperative USS in acute appendicitis based on the histology results post appendicectomy.

## Results

**Figure 1:**
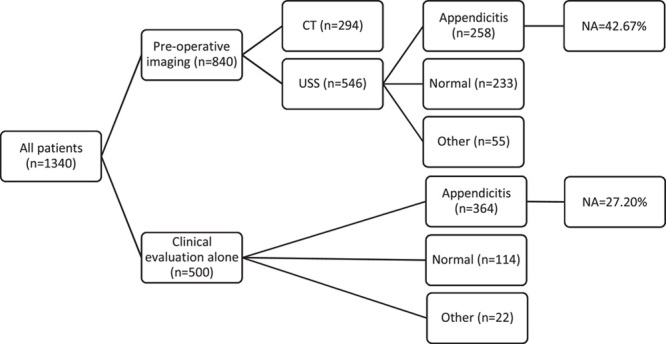
Flow chart showing diagnostic pathway and histological results for the patient groups, including computerised tomography (CT), ultrasonography (USS) and clinical evaluation alone. NA, negative appendicectomy.

The flow chart of this study is shown in Figure 1, summarising their diagnostic pathway and histological results for the two patient groups. Median age and gender are also presented in Table 1. Patients who underwent preoperative CT were excluded in the study.

**Table 1:** Summary of sex and age with the number of laparoscopic appendicectomy performed

Sex	Median age	No. laparoscopic appendicectomy performed
Male	32	449
Female	30	891

Laparoscopic appendicectomy were performed for 500 patients based on clinical diagnosis alone, with 72.8% (*n*=364) confirmed acute appendicitis on histology. Correct diagnosis of acute appendicitis based solely on clinical evaluation is also shown much higher in male patients than female patients, accounting for 71.98% (*n*=262) and 28.02% (*n*=102), respectively.

Other pathologies were also found in histology including carcinoid tumour and uncomplicated faecolith (4.4%/ *n*=22).

22.80% (*n*=114) of the patients without preoperative imaging had negative appendicectomy with 37.72% (*n*=43) of them being female. The above results are shown in Table 2.

**Table 2:** Summary of the number and percentage of the patients underwent laparoscopic appendicectomy without preoperative imaging and their histology results

Histology	Male, *n* (%)	Female, *n* (%)
Normal	71 (20.58)	43 (27.74)
Appendicitis	262 (75.94)	102 (65.81)
Other	12 (3.48)	10 (6.45)
Total	345	155

Preoperative USS were performed for 546 patients who underwent laparoscopic appendicectomy. 12.27% (*n*=67) of them had a positive result of appendicitis and 42.67% (*n*=233) of the total had a normal histology result (negative appendicectomy). The data are shown in Table 3. The sensitivity, specificity, positive predictive value and negative predictive value of USS were then calculated which is presented in Table 4. The NA rate and the *P* value are shown in Table 5.

**Table 3:** Ultrasound and histological results of the patients who underwent USS before laparoscopic appendicectomy

USS results	Normal, *n* (%)	Appendicitis, *n* (%)	Other, *n* (%)
Histology: normal (*n*=233)	166 (48.12)	13 (19.4)	54 (68.35)
Histology: appendicitis (*n*=258)	154 (44.64)	54 (80.60)	50 (37.31)
Histology: other (*n*=55)	25 (7.2)	0	30 (22.39)

USS, ultrasonography.

**Table 4:** Sensitivity, specificity, positive predictive value and negative predictive value of USS in detecting appendicitis

Sensitivity	95% confidence level	Specificity	95% confidence level	Positive predictive value	95% confidence level	Negative predictive value	95% confidence level
25.96%	20.14–32.48%	92.74%	87.90–96.08%	80.60%	70.11–88.03%	51.87%	49.62–54.12%

USS, ultrasonography.

**Table 5:** Summary comparing the negative appendicectomy between the two patient groups

Diagnostic approach	No. patients (*n*)	Histology proven results (*N*)	Negative appendicectomy rate (*P* value)
Clinical evaluation alone	500	364	27.20%
Ultrasound	546	258	42.67% (*P*≤0.00001)

## Discussion

Acute appendicitis is a common cause for acute abdomen with a lifetime of 10% in the UK population. It has always been challenging to have a definitive diagnosis of acute appendicitis due to its nonspecific symptoms and signs which can be mimicked by a variety of pathology^8^. In addition, delay to intervention can increase the risk of complications such as perforation and peritonitis^4^. Clinical decision to perform appendicectomy especially in equivocal cases is common to prevent complications of delayed or missed diagnosis. However NA carries its morbidity and mortality although complications from laparoscopic appendicectomy is generally uncommon^9^.

The patient group with only clinical diagnosis in this study had a NA rate of 27.20% which is consistent with other studies in the literature^10,11^. Poor sensitivity is shown in the patient group who underwent preoperative USS (25.96% with 95% CL 20.14–32.48%), compared with its high specificity of 92.74% (CI 87.90–96.08%)^12^. The poor sensitivity is likely due to its highly operator dependent nature and interoperator variability can exist based on the level of training and experience, which then often leads to laparoscopic appendicectomy due to the clinical uncertainty and potential serious consequences of a missed diagnosis, causing a significantly high NA rate^13^. The use of a standardized reporting system that includes specific criteria for diagnosing acute appendicitis can help to reduce interoperator variability by ensuring that all operators are looking for the same features^13^. Also, a standardized examination technique can also help obtaining the images which are consistent and of high quality. Sex inequality is also shown in this group as the majority of the patients were female, as USS remains a popular choice of its non-radiating property for the patients who is pregnant or in child-bearing age to look for alternative pathology.

Despite advancement in Radiology and ultrasound technique, it remains challenging in evaluating acute appendicitis on USS^14^. This is especially true in our study as more than 85% of the USS examinations cannot visualise the inflammed appendix and hence high NA rate. Interpretation using a five-category system is suggested to improve accuracy (NA rate of 32.4%) and confidence in assessing appendicitis over a traditional binary system^14^. Moreover, the clinical management of appendicitis can be guided through its use and help reducing NA rate^14^.

False negative rate of 44.64% was shown in the preoperative USS group which we as clinicians should be careful and not be misled in interpretating a negative USS result. Alternative pathology could also be responsible for right iliac fossa pain pain such as faecoliths or carcinoid tumour which appendicectomy is also indicated. Other complementary imaging modality such as CT/ MRI can also be used to support an equivocal USS result^15^.

It has been suggested that some clinical features help reducing the radiation exposure or operation risks after a non-diagnostic USS. Indicators include male sex, migration of pain to right lower quadrant, vomiting and leucocytes count greater than 12×10^9^/ l. Probability of acute appendicitis is reduced if less than two indicators present^16,17^. A clinical review in a short-interval after an equivocal USS result can also significantly improve diagnostic accuracy especially in paediatric patients^16,17^.

With an increasing population and healthcare burden, appropriate use of preoperative USS and clinical context are important aspects to be considered for patients presented with right iliac fossa pain. USS may be considered as a better tool in excluding other similar gynaecological pathology. Clinical evaluation remains essential to diagnosis acute appendicitis and reduce NA with its risks and complications. Improvement of clinical evaluation with the use of scoring systems such as Alvarado and Appendicitis Inflammatory Response (AIR) Score can be an option to better guide decision-making in suspected acute appendicitis^18^.

Further research can be also conducted to evaluate the effectiveness of other imaging modalities or diagnostic methods in detecting acute appendicitis. The use of machine learning algorithms or artificial intelligence to aid in the diagnosis of acute appendicitis could also be explored. Additionally, the success rate of current techniques, such as clinical evaluation and ultrasound, should be compared with newer methods and other imaging modality to determine their efficacy, such as CT which had NA rate of 3% in one study ^19^. Finally, more emphasis may be placed on educating healthcare practitioners on the proper use of USS and when it is warranted for the diagnosis of acute appendicitis. This would aid in reducing the number of negative appendicectomies and prevent further complications.

This retrospective study is limited due to its lack of randomisation and selection bias. Preoperative USS data was used without other imaging modalities such as CT or MRI. Further selection bias is involved as only laparoscopic intervention was included with the patients who had open surgery excluded. As laparoscopic surgery is the mainstay intervention for acute appendicitis nowadays, minimal effect is applied.

Moreover observer bias may be present which might have affected the results in favour of clinical diagnosis. There may be a propensity for the sonographers to report a positive USS result with acute appendicitis as their decision may be influenced by the details on the referral form, especially the USS examination was difficult with a variation in appendiceal anatomy and patient body habitus.

## Conclusion

Preoperative USS has been shown to be an less sensitive diagnostic tool in acute appendicitis compared to clinical evaluation alone. When there is a strong clinical suspicion for appendicitis, patients should be managed accordingly even the USS finding is negative. Individual circumstances and surgical implications such as patient factors, financial costs, recovery and potential complications from the surgery should also be taken into account for patients with an unequivocal result undergoing appendicectomy. With an increasing burden of health budget and resources, cautious and appropriate use of ultrasonography would avoid the misdiagnosis and prevent further complications. Thorough clinical evaluation and diagnosis remains an important first step and role in the diagnosis of acute appendicitis.

## Ethical approval

Ethical approval was not required as anonymised data were collected.

## Consent

Consent was not required as anonymised data were collected.

## Source of funding

The authors received no financial support for the research, authorship, and/or publication of this article. This research did not receive any specific grant from funding agencies in the public, commercial, or not-for-profit sectors.

## Author contribution

C.T.K.: study concept and design, data analysis, interpretation and writing the paper. M.A.: data analysis. K.C.: data analysis. M.T.: data analysis. J.Y.: data analysis. S.A.: data analysis. J.R.: data collection, validation and supervision.

## Conflicts of interest disclosure

The authors declared no potential conflicts of interest with respect to the research, authorship, and/or publication of this article.

## Research registration unique identifying number (UIN)

NA.

## Guarantor

Cheuk Tung Kam.

## Provenance and peer review

Not commissioned, externally peer-reviewed.
